# Macrophage Polarization in Chronic Lymphocytic Leukemia: Nurse-Like Cells Are the Caretakers of Leukemic Cells

**DOI:** 10.3390/biomedicines8110516

**Published:** 2020-11-19

**Authors:** Oana Mesaros, Laura Jimbu, Alexandra Neaga, Cristian Popescu, Iulia Berceanu, Ciprian Tomuleasa, Bogdan Fetica, Mihnea Zdrenghea

**Affiliations:** 1 Department of Hematology, Iuliu Hatieganu University of Medicine and Pharmacy, 8 Babes str., 400012 Cluj-Napoca, Romania; ioana.jimbu@umfcluj.ro (L.J.); neaga.alexandra@umfcluj.ro (A.N.); popescu.cristian@umfcluj.ro (C.P.); ciprian.tomuleasa@umfcluj.ro (C.T.); mzdrenghea@umfcluj.ro (M.Z.); 2 Department of Hematology, Ion Chiricuta Oncology Institute, 34-36 Republicii Street, 400015 Cluj-Napoca, Romania; iuliaberceanu@yahoo.com (I.B.); feticab@yahoo.com (B.F.); 3 Department of Infectious Diseases, County Emergency Hospital Alba Iulia, 20 Decebal str., 510093 Alba-Iulia, Romania

**Keywords:** macrophages, CLL, nurse-like cells

## Abstract

Macrophages are terminally differentiated innate immune cells. Through their activation, they can be polarized towards the pro-inflammatory M1 type or the wound healing-associated, anti-inflammatory M2 type macrophages. In the tumor microenvironment (TME), M2 is the dominant phenotype and these cells are referred to as tumor-associated macrophages (TAMs). TAMs secrete cytokines and chemokines, exerting an antiapoptotic, proliferative and pro-metastatic effect on the tumor cells. TAMs can be found in many cancers, including chronic lymphocytic leukemia (CLL), where they are called nurse-like cells (NLCs). Despite the generally indolent behavior of CLL, the proportion of treatment-refractory patients is significant. As with the majority of cancers, despite significant recent progress, CLL pathogenesis is poorly understood. The emerging role of the TME in nurturing the neoplastic process warrants the investigation of macrophages as a significant pathogenetic element of tumors. In this paper, we review the current knowledge on the role of stromal macrophages in CLL.

## 1. Introduction

Every second our body goes through changes. Millions of cells are dying and are being replaced by new ones, especially in the tissues that are most exposed to environmental wear, such as the immune system. To accomplish its function, the immune system increases its turnover during infections, inflammation or tissue damage. The macrophages have a crucial role in initiating the inflammatory response, tissue homeostasis and repair [1,2], but, on the other hand, they play a part in the development of various diseases including cancer [2]. Along with vascular endothelial cells, fibroblasts and other immune cells, a subtype of functionally altered macrophages are part of the tumor microenvironment (TME), setting up the stage for tumor genesis and metastases, promoting genetic instability and tempering anticancer immunity [3]. These types of macrophages are called tumor-associated macrophages (TAMs), and their implication has been proved in different types of cancer [4], including leukemia [5].

Chronic lymphocytic leukemia (CLL) is mostly a disease of the elderlyand is commonly referred to as the most frequent of leukemias in the Western world. According to the World Health Organization classification, CLL, together with its solely tumoral presentation, the small lymphocytic lymphoma (SLL), belongs to the mature B cell neoplasms category, along with other non-Hodgkin’s lymphomas [6].Its manifestations range from mild, in the majority of patients, to aggressive, and the therapeutic approach varies from a watch and wait attitude, to the immediate need for treatment initiation [7]. Over time, a plethora of prognostic markers have been established for CLL, starting with the Rai and Binet staging systems, still in use for almost half a century [8,9], continuing with cytogenetic abnormalities like trisomy 12, del 13q14, del 11q22.3-q23.1, del 17p [10], mutational status of the variable part of the immunoglobulin heavy chain [11], and other recurrent mutations (TP53, NOTCH1, BIRC3, MYD88 etc.) [12,13]. More recently, microRNA profiling, TME characteristics, and the nurturing capacity of TAMs towards leukemic cells have been studied in this regard [2,3,14,15].

TAM infiltration has been associated with outcomes in several cancers including breast cancer, gastric cancer and pancreatic neuroendocrine tumors [16,17,18]. The present review focuses on macrophages’ characteristics in CLL, on the way they influence tumorigenesis, and on their pathogenetic potential and prognostic significance.

## 2. What Are Macrophages?

Macrophages are terminally differentiated innate immune cells. Their surface membrane receptors and cytokine/chemokine production orchestrate their role in homeostasis, initiation and resolution of inflammatory processes, clearance of apoptotic bodies or other debris and last, but not least, tissue remodeling and repair [19,20,21].

The macrophages were described in the late 19th century, by Ilya Metchnikoff, a Russian zoologist, who was intrigued by this type of mobile cells, acting as a sentinel, able to attack, engulf and destroy the invading pathogens [22]. Following the path that the Russian scientist opened, van Furth suggested that tissue macrophages have their origin in circulating adult monocytes [23], coining the term “mononuclear phagocyte system”. It is presently accepted that, based on their origin, there are two types of macrophages: blood monocyte-derived macrophages (MDMs) and tissue-resident ones [24].

Blood monocyte development takes place in the bone marrow, from the hematopoietic stem cells (HSCs), under the influence of growth factors like granulocyte-macrophage colony-stimulating factor (GM-CSF). Once they reach their adulthood, they migrate into the bloodstream and remain there for up to 2 days. During this time, stimulated by specific cytokines and chemokines, they can migrate to different tissues and differentiate into macrophages, providing the restoration of the tissue macrophage pool whenever and wherever it is needed; alternatively, they undergo apoptosis or programmed cell death, consecutively being removed from the circulation [25].

Some of the tissue-resident macrophages do not develop from blood monocytes, but they emerge during the first week of gestation from the yolk sac and migrate to the fetal liver, a primary hematopoietic organ until the bone marrow is ready to overtake this function. They then migrate and populate the respective tissues [26,27]. Tissue macrophages are present in most organs, exhibiting various phenotypes, morphologies, functions and names, according to the anatomic site [24], and have a low turnover during steady-state which is increased during inflammatory processes [1]. Some of the tissue macrophages have an independent self-renewal potential, but the rest of the tissular pool is being subsequently replenished by MDMs [28]. The replenishing of tissue macrophages is regulated by the colony-stimulating factor-1 receptor (CSF-1R), colony-stimulating factor-1 (CSF-1), known as the macrophage-colony-stimulating factor (M-CSF), interleukin-34 (IL-34) and monocyte chemoattractant protein 1 (MCP-1) or C-C motif ligand 2 (CCL-2) [29].

## 3. Activation of Macrophages

**Figure 1:**
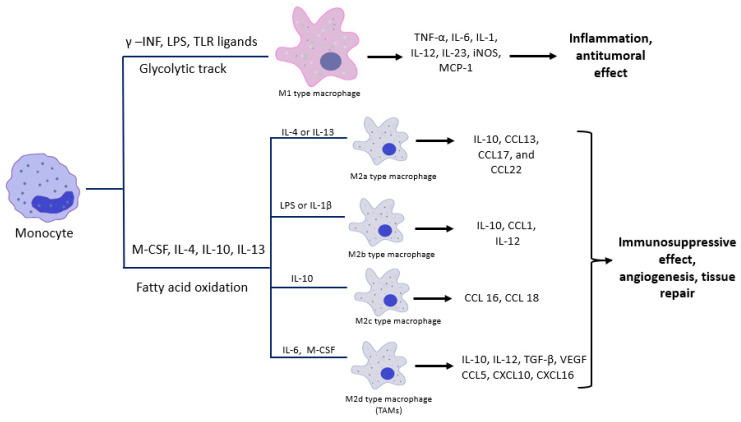
The classical (M1) and the alternative (M2) macrophage activation and their effect on the involved tissues.

When there is a stimulus, like inflammation, infection, debris, or apoptotic bodies, the macrophages are activated and their phenotype changes through the classical or alternative pathways. The outcome of the classical activation will result in the M1 subtype, representing the pro-inflammatory macrophages, and the alternative pathway will lead to the formation of M2 macrophages, which are the anti-inflammatory ones. The paradigm of the M1/M2 subtypes was suggested as a parallel to the T-helper 1 (Th1) and T-helper 2 (Th2) cells [30,31]. Polarization occurs during the macrophage activation process and is controlled by three variables: microenvironment, cytokines and epigenetics [32]. The classical activation of macrophages (M1) takes place under the aegis of Th1 type cytokines like interferon-gamma (IFN-γ) and lipopolysaccharide (LPS), through a glycolytic track [33,34]. Along with the cytokines, transcription factors like the signal transducer and activator of transcription 1 (STAT-1), the nuclear factor kappa B (NF-κB), the activator protein 1 (AP-1), the interferon regulatory factor 5 (IRF-5), influence M1 polarization [35,36,37,38]. After the activation, the cells will express markers like CD68, CD80, CD89 [39,40] and will exert their action by releasing pro-inflammatory cytokines, such as tumor necrosis factor-α (TNF-α), IL-6, IL-1, IL-12, IL-23, inducible nitric oxide synthase (iNOS), MCP-1 (Figure 1) [41].

The alternative pathway (M2) is triggered by various cytokines, the most important being IL-4 and IL-13, which are Th2 type cytokines, and is, energy-wise, fueled through fatty acid oxidation [33,34]. M2 macrophages were further classified into four subcategories: M2a, M2b, M2c [30,42] and M2d [43,44]. M2a is triggered by the effect of IL-4 and IL-13 and releases cytokines like IL-10, CCL13, CCL17, and CCL22, M2b is activated by LPS or IL-1β and produces IL-10, CCL1, IL-12, M2c is stimulated by Il-10 and discharges CCL 16 and CCL 18 chemokines. The M2d type is credited by some authors as TAMs, is stimulated by IL-6, M-CSF and the activation of the adenosine receptors and releases high amounts of IL-10, TGF-β, VEGF, CCL5, CXCL10, CXCL16 and low levels of IL-12 (Figure 1). M2 activation also depends on transcription factors, such as STAT-6, IRF-4, hypoxia inducible factor-1 alpha (HIF-1α), peroxisome proliferator-activated receptor gamma (PPAR-γ), CCAAT/enhancer-binding proteins (C/EBPβ) [45,46,47,48,49]. The immunophenotyping of M2 macrophages reveals the presence of markers such as CD64, CD163, CD200, CD206, CD209 [39,40,50].

In contrast to M1 macrophages, the M2 subtype exerts, as mentioned before, a healing effect and will release cytokines into circulation like vascular endothelial growth factor (VEGF), transforming growth factor-β (TGF-β), epidermal growth factor (EGF), and arginase [1,14]. Furthermore, M1 cells produce iNOS, which stimulates the production of nitric oxide (NO), while the M2 subtype produces TGF-β, an NO inhibitor. Additionally, M1 polarization uses STAT-1 as a transcription factor, while M2 polarization uses STAT-6. These two pathways are mutually exclusive.

The complete mechanism of macrophage polarization is not yet fully understood, but in vitro studies on human macrophages have shown that serotonin or 5-hydroxytryptamine (5-HT) may also have an important role in this activation process [51]. This substance is known for its role as a neurotransmitter, but, in recent years, its immunomodulatory role has been discovered. Most of it is secreted by the enterochromaffin cells in the intestine, transported into the bloodstream, collected by the platelets and released during their activation (thrombus, inflammation) [52]. As an immunomodulatory substance, it coordinates the cytokine discharge from the macrophages and monocytes, suppressing TNF-α and IL-1β and promoting the macrophage polarization towards M2 subtype, thus stimulating the growth and regeneration of the tissue [53].

The M1 and M2 pathways are antithetic: while one destroys, the other repairs and an imbalance between these pathways could lead to the appearance of autoimmune diseases, metabolic instability and even cancer. This dichotomous model, proposed in 2000 by Charles Mills [31] has since evolved to accept the occurrence, in vivo, of a continuum of macrophage phenotypes situated between the two polarized states [54].

## 4. The Macrophages and Cancer

The TME contains a plethora of immune cells, including macrophages (~50%) [55], which are attracted by high amounts of lactic acid and certain cytokines and subsequently converted into TAMs [56]. Occasionally, these cells are regarded as equivalent to M2 macrophages, but some authors have reported that even though TAMs mostly have M2 characteristics, they may also share some of the M1 phenotype’s functions [57]. The heterogeneity and plasticity of TAMs allow them to satisfy the need of any tumor, receiving “instructions” based on the tumoral tissue’s type [58]. In some types of neoplasia, the high rate of TAMs infiltrating the TME correlates with a worse disease outcome, as TAMs release cytokines that promote the immune escape, the anti-apoptotic effect, the growth, the metastasis and the chemoresistance of the tumor cells [59].

TAM differentiation is orchestrated by cytokines released from the stromal and tumor cells [60]. The accelerated tumor metabolism and growth, without the required vascular sustenance, generates hypoxia. Thus, along with lactic acid, chemokines including, but not limited to CSF-1, CCL-2, CCL-5, VEGF, are released, and macrophages are attracted to the TME [56,61]. Here, under the action of IL-6, IL-10, IL-12, TNF, CCL-2, CCL-5, CSF-1and the overexpression of epidermal growth factor receptor (EGFR), macrophages undergo an M2 like polarization and become TAMs [62,63]. Several murine and human studies have proven that the depletion of CSF-1, CCL-2, VEGF and EGFR lowered the TAMs infiltration, and, implicitly, tumor progression and metastasis [63,64,65]. Cytokines like TNF-α, IL-1β, IL-6, IL-8, IL-10, TGF-β, stimulate epithelial–mesenchymal transition (EMT) [66,67,68,69,70,71], by which tumor cells gain multi-directional differentiation and self-renewal potential, and the ability to migrate and invade tissues, creating an ideal background for treatment resistance in tumors [72,73].

## 5. TAM-Related Cytokines and Chemokines

### 5.1. TNF-α

TNF-α is largely produced by macrophages and exerts its role as an inflammation mediator. Usually, TNF-α through its receptors, TNFR-1 and TNFR-2, activates Janus kinase (JAK) and NF-κB signaling pathways and further regulates cell proliferation, survival, differentiation and, finally, apoptosis [74]. When it comes to neoplasia, TNF-α has an ambivalent role, as it could be both an enhancer and a tamer of cancer. Studies have proven that at high doses, TNF-α induces tumor cell apoptosis and stimulates anti-tumor immunity, while at low doses, it helps tumor growth, metastasis and the EMT, in both murine and human models [75,76]. However, the exact mechanism of action is still debatable, but it could involve the TNFR-1 and TNFR-2 distribution, and, of course, the tumor location and stage [77,78]. In CLL, TNF-α is constitutively produced by the CLL B cells and carries an antiapoptotic role and sustains tumor proliferation. Higher levels of TNF-α have been correlated to more aggressive disease [79,80].

### 5.2. IL-1β

IL-1β, also known as mononuclear cell factor (MCF), lymphocyte-activating factor (LAF) or endogenous pyrogen (EP) [81] is another cytokine with divergent activity. Commonly it is considered to be one of the most powerful enhancers of inflammation, as it stimulates the innate immune cells and promotes the polarization of T cells towards Th1 and Th17 phenotypes, exerting its role in adaptive anti-tumor immunity. However, released by the TAMs in the TME, IL-1β has an opposite role, supporting EMT, tumor growth and anti-apoptosis [82,83,84]. As well as TNF-α and IL-6, IL-1β exerts it pro-tumoral function through the NF-kB and mitogen-activated protein kinase (MAPK) and other signaling pathways like protein kinase B (AKT) or Wingless (WNT) [85,86,87]. High levels of IL-1β are correlated with bad prognosis [88].In the context of CLL, IL-1 is produced by the B CLL cells and induces differentiation and activation of neoplastic cells [89]. However, in the serum of CLL patients, high levels of IL-6 were associated with low levels of IL-1β, which seems to lead to the development of tumor cells [90].

### 5.3. IL-6

IL-6 is a cytokine produced by the immune cells, especially by macrophages. By binding the IL-6R and activating STAT 3 phosphorylation, it exerts its pro-tumoral effects (anti-apoptotic, immunosuppression, angiogenic, metastatic) [91] and apparently, it promotes EMT, as well [69,92], possibly promoting the tumor development and self-renewal of malignant cells. IL-6 and cancer stem cells stimulate one another. For example, in glioma, via the MYD88-TLR4 pathway, glioma stem cells stimulate the TAMs to produce IL-6, which, in turn, will stimulate the EMT via the JAK/STAT3/Snail pathway. This positive feed-back trail will support and nurture glioma development [93]. Higher levels of IL-6 have been correlated to worse disease outcomes [94]. To our knowledge, in CLL, TAMs do not produce Il-6, but in turn they strongly influence its autocrine production by the CLL cells [95,96]. In CLL, IL-6 can be correlated to a poor disease outcome, as it holds a pro-tumorigenic effect as well [97].

### 5.4. IL-10

IL-10 is an anti-inflammatory cytokine, mainly secreted by macrophages, but also by neoplastic cells, in hypoxia conditions [98]. IL-10 exerts its role by binding to its receptor and blocking the anti-tumor immunity, inhibiting pro-inflammatory cytokines like IFN-γ, IL-6, IL-8, IL-12, IL-1β and TNF-α, suppressing the M1 polarization, and restraining the major histocompatibility complex II (MHC II) molecule on the surface of the macrophages [99]. It also activates STAT3 phosphorylation, promoting anti-apoptotic, immunosuppressive, angiogenic and metastatic reactions [100]. IL-10 protects the tumor, while inhibiting the chemotaxis of neutrophils, monocytes and dendritic cells in the TME, by suppressing the CC and CXC chemokines [101]. Promoting EMT is another way of nurturing the tumor. In vivo studies on mice models have shown that IL-10 seems to be one of the pillars of EMT and of obtaining the stemness of tumor cells [102,103]. Treatments based on inhibitory molecules of IL-10 are currently being studied. In CLL, IL-10 is also associated with treatment resistance and immune suppression. This cytokine has an important role in the resistance of CLL cells to ibrutinib. Apparently, ibrutinib targets Bruton’s tyrosine kinase from the surface of TAMs from the CLL microenvironment, modifying their function and stimulating them to produce IL-10, providing the tumor cells with resistance to treatment [104]. B CLL cells also produce IL-10, through the stimulation of B-cell receptor (BCR), in murine and human studies [105].

### 5.5. TGF-β

TGF-β is a cytokine with a dual role in tumor evolution, promoting and inhibiting it as well. There are three types of TGF-β, but TGF-β1 is the most often implicated in tumorigenesis. The TGF-β ligands exert their role through binding TGFβR1 or TGFβR2 [106]. In the incipient tumor stage, it acts as a tumor suppressor, by inducing apoptosis, inhibiting the tumor cell proliferation and favoring their differentiation towards healthy cells. In late tumor stages, it has a tumor promoting role, stimulating EMT, immunosuppression, angiogenesis, tumor cells self-renewal and chemo-resistance [107,108]. Using TGF-β as a target in cancer treatment is still controversial, as it needs further clinical trials [109]. In CLL patients’ serum, large quantities of TGF-β are found, but despite this, the neoplastic cells develop a resistance to this cytokine’s pro-apoptotic effect [110]. Further, the leukemic cells produce TGF-βthat stimulates the growth of TAMs, which will nurture the tumor itself [111].

### 5.6. CCL2

CCL-2 or MCP-1 is a chemokine from the C-C chemokine family, with an important role in recruiting macrophages to the TME and EMT. Tumor cells are the main source of CCL-2, but it can be also secreted by non-tumor cells [112]. By binding CCR-2, CCL-2 promotes tumor growth and metastasis. In vitro studies on human lung squamous cell carcinoma and lung adenocarcinoma cells have correlated the high levels of CCL-2 with worse disease prognosis [113]. The inhibition of the CCL2–CCR2 complex or CCL2 or CCR2 alone, has provided a lowering in tumor growth and prolonged survival in some cancers, giving another opportunity to evaluate a new cancer treatment [114,115]. The CCL2 level is high in the serum of CLL patients. The presence of this cytokine will help create a microenvironment that will be able to protect the tumor cells against apoptosis and will stimulate its progression [116].

## 6. Macrophages in CLL

CLL is a heterogenous hematologic disease, characterized by the accumulation of mature lymphocyte-like malignant cells in the bone marrow, peripheral blood, lymph nodes (LN) and spleen. Usually, it behaves as a mild, slowly progressive disease, but in a proportion of cases, a more aggressive, sometimes violent evolution and chemoresistance are observed. Research has focused on finding the background for CLL cells increasing their proliferation rate, enhancing their anti-apoptotic features, and their resistance to treatment. Studies on in vitro cultured CLL cells have shown that the accelerated proliferation and the low rate of apoptosis are not independent cell abilities, but are linked to the TME [117,118]. Hence, a closer look to the CLL TME was necessary, which led Burger et al. to observe that the CLL microenvironment contains a population of mononuclear cells exhibiting common features with the thymic nurse cells, and release mediators that prevent the apoptosis of CLL cells in vitro [111]. Later in vivo and in vitro studies in CLL patients have proven that these cells can be found in the secondary lymphoid tissues and that they are TAM correspondents in CLL [119,120,121,122]. TAMs can be observed in different types of solid tumors, holding particular phenotypes and producing cytokines and chemokines according to the lineage of the neoplastic cells. They are usually correlated with a worse disease outcome. Such cells are encountered in leukemias as well, where they are called leukemia-associated macrophages (LAMs) [14]. The LAMs in CLL are known as nurse-like cells (NLCs) [111]. They are positively correlated with peripheral blood monocytes and serum beta 2 microglobulin levels [15] and their abundance was linked to an adverse prognosis, like in the case of other TAMs [123].

NLCs derive from blood monocytes under the influence of CLL cells and in return, they release chemokines, like C-X-C motif ligand 12 and 13 (CXCL12 and CXCL13) to attract even more CLL cells [111]. They have an M2 like phenotype, expressing on their surface, markers like CD14, CD11b, CD68, CD163 and HLA class II [124]. Once in the right place, NLCs support CLL cells by enhancing their proliferation, survival, invasion and metastasis, and by disrupting the anti-tumoral immunity.

## 7. NLC Functions

### 7.1. Recruiting Cells

The CLL cells express functionally active chemokine receptors such as CXCR3, CXCR4 and CXCR5 that enable their migration and homing process. Normal B cells usually express CXCR4 and CXCR5, while CXCR3 is found on a small number of CD5+ and negative B lymphocytes.

CXCR3 is expressed by CD4^+^ CD8^+^ and activated T cells and by NK cells, binds its ligands CXCL9, CXCL10, CXCL11 and helps trafficking activated T lymphocytes, dendritic and NK cells to the inflammation site. During homeostasis periods, CXCL9, CXCL10, CXCL11 are produced in small quantities by endothelial cells, fibroblasts and monocytes, with their secretion being up-regulated under the effect of IFN-γ and TNF-α [125,126,127].

**Figure 2:**
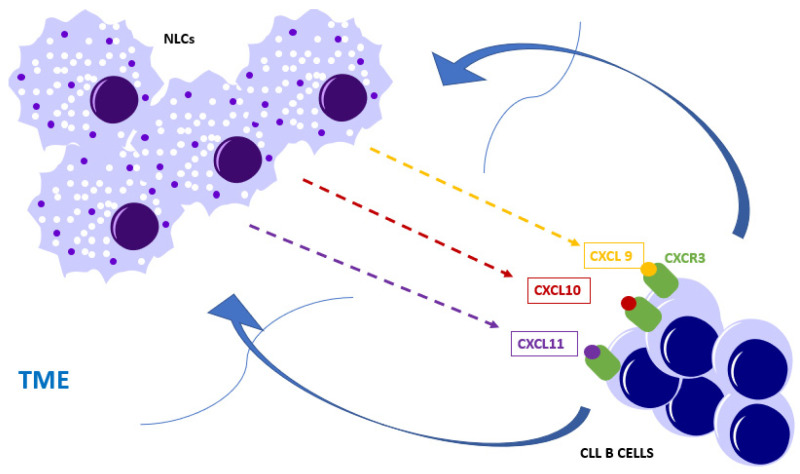
Nurse-like cells (NLCs), along with endothelial cells, fibroblasts and monocytes, secrete chemokines like CXCL9, CXCL10, CXCL11, which will bind the CXCR3 receptor expressed on the chronic lymphocytic leukemia (CLL) B cells and attract the cells into the tumor microenvironment (TME).

In CLL, chemokines CXCL9, CXCL10, CXCL11 are produced by TME components, including NLCs [125,127]. The axis CXCR3-CXCL9/CXCL10/CXCL11 has a chemotactic role for CLL cells (Figure 2) [120]. CXCR3 expression levels are variable and low levels are associated with advanced disease and poor prognosis in CLL [128].

CXCR4 is expressed on both CLL B cell-like and normal B cells, but its expression appears to be five times higher in the former [129]. CXCR4’s ligand is called stromal cell-derived factor-1 (SDF-1) or CXCL12 and is a chemokine secreted by the bone marrow stromal cells, under the control of TGFβ1 and HIF-2α. The CXCR4–CXCL12 axis influences the homing in the bone marrow of the CLL cells and their interactions with stromal cells, favoring their adhesion to the stroma, and thus their chemoresistance [111,130]. NLCs also secrete CXCL12, enabling the chemotaxis of CLL cells through the endocytosis of CXCR4 [119,129], a process that will imply the down-regulation of the CXCR4 expression level after the migration [111]. High expression of CXCR4 is correlated with an advanced stage of CLL [131].

CXCR5 is usually expressed on the surface of mature B cells, a small fraction of T cells, and a type of skin-derived dendritic cells [132]. Its ligand is known as CXCL13 or B-cell-attracting chemokine 1 (BCA-1) and is secreted by the stromal cells within the lymphoid follicles. The axis CXCR5–CXCL13 stimulates the recruitment of naïve B cells within the germinal center of the lymphoid follicles. As the CLL B cells highly express CXCR5, through a chemokine gradient, they are attracted by the secretion of CXCL13, from the tumor stromal cells and NLCs. The binding of CXCR5 and CXCL13 stimulates the endocytosis of the receptor, the formation of actin polymers, the activation of ERK1/2 (extracellular signal-regulated protein kinases 1 and 2) and the migration and homing of the CLL cells to the LNs, where they are defended against apoptosis [125,133,134].

Apart from these, in vitro studies have revealed that NLCs co-cultured with purified CD19+ CLL cells, induce, through B-cell receptor (BCR) activation, the production of T lymphocytes-specific chemokines: CCL 3 and CCL 4. Through their receptors, CCR 1 and CCR 5, they recruit T cells and other leukocytes, including some NLCs precursors. The CCL 3 and CCL 4 levels correlate with the zeta-associated protein 70 (ZAP-70) expression on the CLL cells surface [135].

### 7.2. Enhancing Survival and Antiapoptotic Effect

It can be asserted that NLCs sustain and improve CLL cells survival, as it was observed that patients with lower NLCs levels have better overall survival [120]. Through the activation of the BCR and NF-kB pathways, NLCs exert an antiapoptotic effect on CLL cells, enhancing their survival [136].

As the BCR pathway is initiated, enzymes and adaptor molecules, including the spleen tyrosine kinase (Syk), are activated and phosphorylate Bruton tyrosine kinase (BTK) and phosphoinositol-3 kinase (PI3K). The phosphorylated BTK and PI3Kwill eventually lead to the activation of NF-kB, RAS and MAP kinases pathways [125,137], which will impair the anti-tumoral immunity, providing an antiapoptotic effect and enhancing the survival of CLL cells.

Another way of activating the NF-kB pathway is through the release of B cell activating factor (BAFF) and a proliferation-inducing ligand (APRIL), by the NLCs and other stromal cells [138]. BAFF and APRIL are both TNF family members and are usually expressed by monocytes, macrophages, dendritic cells and T cells. They bind BAFF-R, TNFR homolog transmembrane activator and CAML interactor (TACI) and B cell maturation antigen (BCMA) receptors found on B cells [137], and finally, will activate downstream NF-kB pathways.

CD40L (ligand), another TNF family member, is usually expressed on the surface of T cells. In a physiological context, the CD40L would bind the CD40 receptor expressed on the B cells and would regulate cell differentiation [137]. However, CLL B cells possess an aberrant expression of CD40L, which promotes CLL cells survival and differentiation. The aberrant expression of CD40L is up-regulated by the presence of APRIL and BAFF, which are stimulated by the activation of the CCL2–CCR2complex [125,138]. CCL2 gene is highly expressed on NLCs [120]. Although it appears not to have a direct effect on CLL cells, the CCL2–CCR2 complex attracts more monocytes to the TME and stimulates the NLCs to release factors like BAFF and APRIL, building up the anti-apoptotic effect, tumor progression and metastasis [111,139].

CD38 and ZAP-70, members of the Syk family, are both correlated with a bad outcome for CLL patients [140]. ZAP-70 is usually present on the surface of T and NK cells, but not on normal B cells. However, CLL cells also possess this marker, and it appears that CD 38 binds with CD31 ligand, expressed by NLCs and leads to ZAP-70 phosphorylation, which will further activate the BCR and through signaling pathways like PI3Ks and BTK will enhance CLL cells proliferation and survival [141].

Another important cytokine involved in immune suppression and CLL cell survival is IL-10. In vitro studies on CLL patients’ serum and in vivo studies on mice models have revealed that IL-10 is found in larger quantities in CLL patients’ serum, than in healthy controls [105,142]. IL-10 is a suppressor type 2 cytokine that inhibits MCH class II on the surface of APC, limiting the antitumoral immunity [143]. Apart from the CLL cells chemotaxis mentioned above, CXCL12-CXCR4, regulates the levels of IL-10, through STAT3 pathway. In vitro studies on CLL patients’ serum have revealed that inhibition of STAT3 phosphorylation down-regulates IL-10 and reinforces anti-tumoral immunity [144].

After inhibiting the above mentioned antiapoptotic factors in co-cultures of CLL cells, some of these cells survive, meaning that they have other ways of evading the apoptosis. Based on this assumption, in 2018, Abbaciet al. published an article about the effect of brain-derived neurotrophic factor (BDNF) on CLL cells. BDNF’s expression is high on the surface of CLL cells and by binding the neurotensin receptor 2-tropomyosin-related kinase receptor B (NTSR2-TrkB) complex which is highly expressed on CLL cells surface as well, activates B-cell lymphoma 2 protein (BCL-2) and other signaling pathways, leading to an anti-apoptotic effect [145]. Recent in vitro studies on CLL patients’ cells suggest that BDNF is in fact secreted by the NLCs [146].

Apart from secreting and stimulating cytokine and chemokine production, NLCs may also provide an antiapoptotic effect through their direct interactions with the CLL cells. CD2 expressed on the surface of NLCs, binds the lymphocyte function-associated antigen 3 (LFA-3) or CD 58, expressed on CLL cells, inhibiting their programmed death. It also seems that LFA-3 produces soluble LFA-3 (sLFA-3), which may inhibit the T-cell immune response, once again helping the CLL cells to evade immune control. In a study which included 60 chemoimmunotherapy-treated CLL patients, the levels of sLFA-3 were correlated with shorter overall survival [147].

### 7.3. Proliferation Stimulation

CLL cell proliferation occurs in the pseudo-follicles, which are proliferation centers found in the LNs and BM that contain lymphocytes and prolymphocytes. In the LNs, the CLL cells have the first contact with antigens and also receive proliferation and antiapoptotic signals [148,149,150].

The proliferation rate of CLL cells depends on the cell characteristics, but it seems that, in a small part, NLCs have some implication in this process. For example, CD38^+^ cells have a higher Ki-67 expression [151] and proliferate much faster than CD38^−^ cells [152]. CD38 is a membrane protein, with a higher expression in the BM and secondary lymphoid organs [153], and, as mentioned before, represents a negative prognostic factor for CLL patients. CD38 binds its ligand, CD31, which is expressed by the stromal cells and NLCs [153,154] and stimulates the ZAP-70 phosphorylation and further on, it activates BCR and other signaling pathways that will lead to the proliferation of CLL cells [141,155]. The presence of CD38 and ZAP-70 appears to be synergic [154].

## 8. Conclusions

TAMs, known as NLCs in CLL, are part of the TME and resemble M2 polarized macrophages, witha pro-tumorigenic effect, while the M1 subtype holds a pro-inflammatory, anti-neoplastic effect. In some types of neoplasia, the TAM infiltration level was correlated with a dismal prognosis, but this was not yet proved when it comes to CLL. We know that isolated in vitro cultured CLL cells die, but co-cultured with the NLCs, they are able to proliferate, suggesting that a clue for CLL cure may lie not only in the tumor cell characteristics, but in the particularities of the TME as well. A better understanding of the TME could allow for the development of new and more effective antineoplastic therapies targeted at its modulation. This could be a key element towards a personalized cancer therapy, with better tolerance and less adverse effects, compared to the classical chemotherapy approach.

The road is indeed arduous and long, but the promise that we will be able to personalize the treatment for diseases such as CLL, to expand the overall survival and the treatment-free survival for these patients should provide enough fuel to carry on the research and enrich the knowledge we have obtained so far.
